# Exploration of the Adsorption Reduction of the Pigment Aggregates Strength under the Effect of Surfactants in Water-Dispersion Paints

**DOI:** 10.3390/polym14050996

**Published:** 2022-02-28

**Authors:** Antonina Dyuryagina, Aida Lutsenko, Kirill Ostrovnoy, Vitaliy Tyukanko, Alexandr Demyanenko, Meiramgul Akanova

**Affiliations:** Department of Chemistry and Chemical Technology, M. Kozybayev North Kazakhstan University, Petropavlovsk 150000, Kazakhstan; adyuryagina@inbox.ru (A.D.); l-a.13@mail.ru (A.L.); kostrovnoy@mail.ru (K.O.); demianenkoav@mail.ru (A.D.); meiramgul-87@mail.ru (M.A.)

**Keywords:** adsorption, water-dispersion paints, acrylic film-forming agent, disaggregation, titanium dioxide, surfactants, organic coatings

## Abstract

This article presents the results of studying the disaggregation of titanium dioxide in water-dispersion compositions based on an acrylic film-forming agent under the action of a surfactant. The possibility of using polyether siloxane copolymer (PC) and sodium polyacrylate (NaPA) in paint and varnish compositions as modifying additives of the dispersing effect is proved. The correlation between the dispersing effect of surfactants and the amount of their adsorption on the pigment is proved. NaPA, which provides a greater reduction in adsorption strength, demonstrates a greater dispersing effect than PC. It was found that the larger the size of the aggregates of pigment particles, the greater the disjoining pressure created by the surfactant. An equation is derived that generalizes the cumulative contribution of surfactant concentration and the content of the film-forming agent in suspensions to the average particle diameter of pigment. The introduction of NaPA in the amount of 0.25 g/dm^3^ into the paint allows the rate of acid corrosion to be reduced by 2 times, the number of pores in the coating to be decreased, and the adhesion of the coating to be significantly increased (by 2 points according to ISO 11845: 2020).

## 1. Introduction

An analysis of the current range of paint and varnish products shows that traditional organo-soluble paint and varnish materials that have a harmful effect on humans and pollute the atmosphere are gradually being replaced by progressive ones that do not contain organic solvents or contain them in limited quantities [1]. These include water-diluted, water-dispersion, and powder paint materials, and solvent-free systems.

The rapid progress of water-dispersion paint materials from the group of water-based paint materials is due to a number of their advantages in comparison with other paint materials from the environmentally-friendly category. They provide the possibility of low-temperature drying up to room temperature, and, unlike others as well as materials with a high dry residue, completely exclude the use of organic solvents [2,3].

The process of combining a solid component—a pigment with a liquid polymer medium in the manufacture of paint coatings is very important, since it affects the technological and painting properties of paints, as well as many operational properties of coatings. The main thing in this process is the interaction of the pigment with the polymer at the interface of the solid and liquid phases, since its intensity determines the dispersion of solid particles in this medium and the nature of the resulting combined structure “solid phase–polymer”, which determine the subsequent properties of this materials. This was established by the works of academician P. A. Rebinder on structure formation and physicochemical mechanics of dispersed systems [4] and further developed by the employees of his school—A.B. Taubman and S.N. Tolstoy [4,5,6]—who studied the interaction of pigments with polymers at the interface for a wide range of models and real objects. One of the essential factors determining the effectiveness of protective, structural–mechanical, and decorative properties of paint coatings (opacity, hardness, porosity, tear and impact strength, adhesion) is the degree of dispersion of the pigments included in their compositions [1]. The dispersion of pigments in the aqueous medium of the film-forming agent is one of the main and energy-intensive stages of the production of water-dispersion paints, and thus much attention has been paid to the improvement of the dispersion technology. The amount of energy for the destruction of solid substrates, in particular pigments, depend on a large number of factors. 

The optimal condition for dispersion is the maximum reduction of the interfacial surface energy at the interface between the solid pigment and the liquid medium, which is achieved by using surfactants. The introduction of surfactants into water-dispersion compositions opens up the possibility of purposeful changes in the structural layers formed at the interphase boundary “pigment-dispersion medium” and, as a result, allows fine-tuning of the process of disaggregation.

The mechanism of the disaggregating action of surfactants is based on the Rebinder effect [4], which consists of an adsorption decrease in the strength of pigment aggregates when exposed to surfactants. Surfactants easily penetrate from the liquid phase in which the dispersion is carried out into the internal cavities (micro cracks) of aggregates of pigment particles to the obstacle caused by the proper size of the adsorbed molecules. The adsorption of surfactants leads to the appearance of a positive disjoining pressure in the direction of the development of micro cracks, which favors the increase in deformations and mechanical separation of aggregates. At the same time, an improvement in the wetting of pigments with resin (varnish) solutions is recorded [7,8].

It is known that surfactants are used in paints as additives of the dispersing effect. Surfactants in these systems can be low molecular weight or oligomeric compounds [9,10], acrylic copolymers containing (meth)acrylic acid or their derivatives [11]. Copolymers of polyether and siloxane, polyphosphates (mainly in matte latex paints), salts of oligocarboxylic acids, copolymerization products of unsaturated polyethers, and unsaturated carboxylic acids or their anhydrides are widely used [12].

However, the effect of surfactants is very specific and depends on their qualitative and quantitative composition, the properties of the system components, and the nature of the interaction between them.

The aim of our work was to explore the influence of various factors on the processes of adsorption reduction of the strength of pigment aggregates. This required solving the following tasks:

1. To explore the surface-active properties of additives at the interfacial boundaries with air and a solid surface.
2. To study the regularities of the development of the processes of disaggregation of titanium dioxide (grade R-02) in water-dispersion compositions based on acrylic film-forming agent as a result of the adsorption action of surfactants, namely polyether siloxane copolymer and sodium polyacrylate.
3. To optimize the compositions of water-dispersion paints by the content of film-forming agent and surfactant using probabilistic–deterministic modeling methods to ensure maximum pigment disaggregation.

## 2. Materials and Methods

### 2.1. Materials

**Figure 1:**
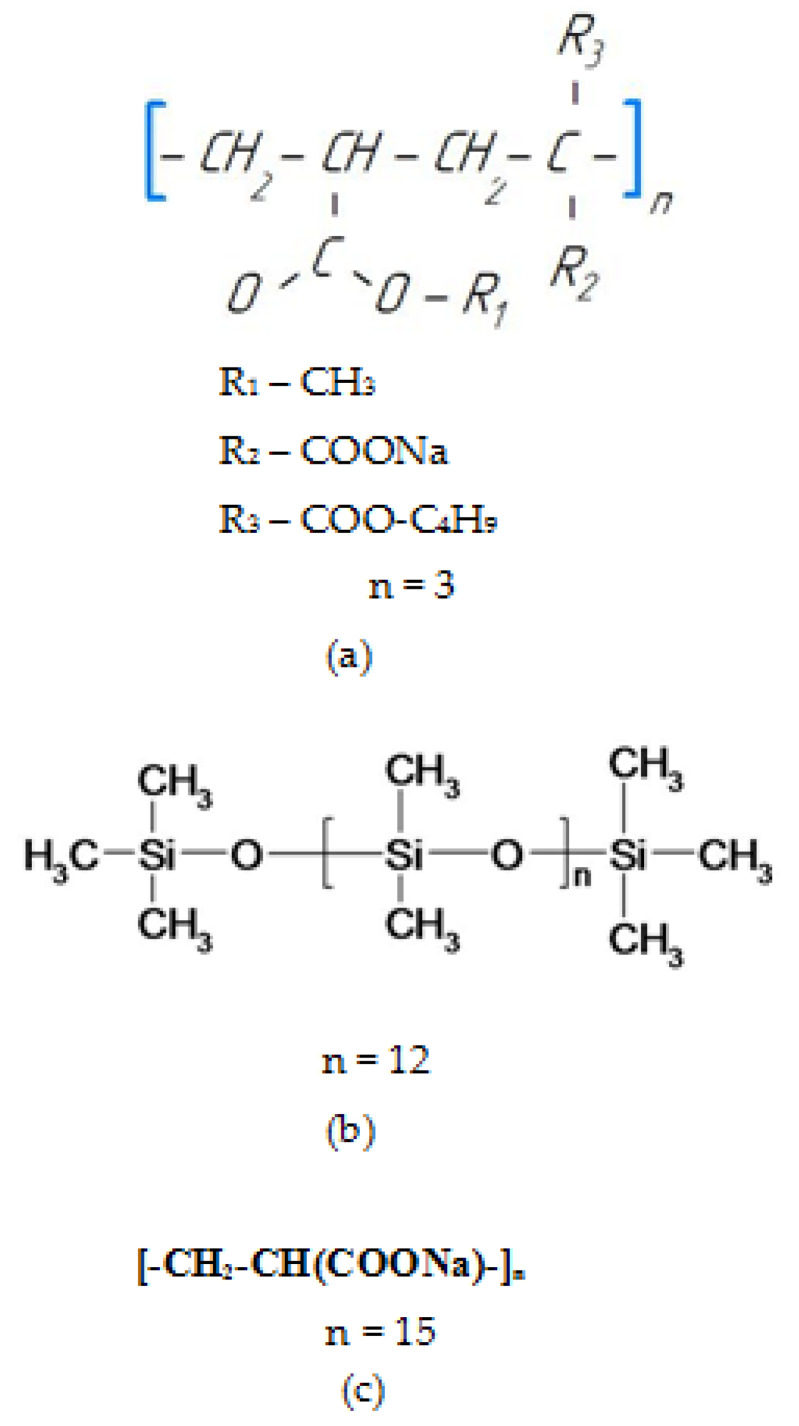
Formulas of materials used: (**a**) acrylic varnish, (**b**) PC, (**c**) NaPA.

Acrylic varnish "Platinum", brand "Grand Victory", produced by NANOTECH PAINTS LLP (Almalybak village, Almaty region, Kazakhstan) was used as a polymer (the formula is shown in Figure 1a). Distilled water was used as a solvent. As a pigment, was used titanium dioxide grade R-02 (GOST 9804-84) of the rutile form (mass fraction of titanium dioxide of the rutile form—95%), produced by PJSC “Crimean TITAN”, Armyansk, Ukraine.

The surfactants used were as follows:

(1) Industrial additive for TEGO water-based paints, Glide 100 brand (hereinafter referred to as PC), which is polyether siloxane copolymer (the formula is shown in Figure 1b), produced by Evonik Operations GmbH, Essen, Germany.
(2) Industrial additive for water-based paints TEGO, brand Dispers 715w (hereinafter NaPA), which is sodium polyacrylate (the formula is shown in Figure 1c), produced by Evonik Operations GmbH, Essen, Germany.

### 2.2. Computer-Micro-Optical Method for Determining Dispersion Parameters 

In M. Kozybayev North Kazakhstan University (Petropavlovsk, Kazakhstan) was developed a computer-micro-optical complex for the analysis of powders and suspensions [13]. To study the regularities of the dispersion of titanium dioxide in water-dispersion compositions, the method of computer-micro-optical analysis based on the application of the complex [13] was developed. This method includes computer fixation of suspensions and their mathematical processing. This method provides a fast, object-oriented, and reproducible analysis of the particle size distribution of the suspension in an automatic mode. The dispersion of the pigment was controlled by changing the average particle diameter of the pigment (APDP) in microns and the content of fine fractions (CF, %). A description of this method is attached to the manuscript as Supplementary Materials.

This method has been successfully applied by the authors to estimate the size distribution of pigment particles in suspensions of pentaphthalic, chlorvinyl, uralkid, and silicone paints [14].

### 2.3. Sample Preparation

The method of preparation of suspensions based on an acrylic film-forming agent consisted of preliminary dissolution of a fixed mass of TiO_2_ pigment (3.75 %) in a solution of film-forming agent (10–30%), followed by dosing of the studied surfactants (0–1 g/dm^3^ per pigment mass). The suspensions were prepared at a temperature of 20 °C in a sealed reactor (volume 0.2 dm^3^, filling factor 0.60), equipped with a mixing device (impeller agitator, rotation frequency 300 min^−1^). The suspensions were mixed for 30 min until the full completion of the adsorption processes and the formation of a homogeneous mass stabilized by the degree of disaggregation of the pigment. To stabilize the deformation processes in the films, the test samples of paint and varnish suspensions were placed on a slide using a pneumatic dispenser (drop volume 0.02 mL), then fixed with a cover glass and kept under static load (15 g/cm^2^) for 10 min.

### 2.4. Adsorption Measurement

Control over the development of the adsorption process was carried out by measuring the surface tension of the liquid phase at the interface with air according to the standard ring tear-off method (the Du Noüy ring method [15]). The maximum force of ring tear-off was measured using a model DST tensiometer.

The effect of surfactant concentration (CS, g/dm^3^: 0–1), temperature (T, °K: 293–313), as well as the content of the film-forming agent (acrylic derivative) (CAD, %: 0–30) was explored. The duration of the experiments in all cases was 30 min, which, according to preliminary studies, was sufficient to achieve equilibrium states. Upon completion of the operations, the components of the suspension were quickly separated by centrifugation, at the same temperature conditions at which the experimental studies were performed. The amount of adsorbed surfactant (A, g/g pigment) was determined according to that previously obtained for the corresponding compositions of film-forming agent calibration curves (σ = *f* (CS)). The determination of the surface tension values for the surfactant concentration was carried out 5 times.

The quantity of adsorbed additives on the solid surface of titanium dioxide was determined from the difference between their equilibrium concentrations in the solution before and after adsorption, at a fixed mass of pigment (m = 0.4 g) and a constant volume of the solution (V = 0.25 dm^3^).

### 2.5. Planning an Experiment Using the Method of Probabilistic–Deterministic Planning

Modeling of the adsorptive effect of surfactants on the process of dispersing pigments was carried out within the framework of the method of probabilistic deterministic planning (PDP) [16]. Research work using PDP consisted of several stages:

1. Determination of factors and levels of their variation.
2. Constructing an experiment plan in the form of a plan-matrix consisting of m columns corresponding to the number of input parameters (factors), and n rows corresponding to the number of variations (the number of experiments) of the given levels (numerical value) of factors. To ensure the orthogonality of the design matrix, each level of one input parameter was set only once with each level of any other input parameter.
3. Conducting an active experiment according to the generated plan-matrix and establishing the numerical values of the response function (output parameter).
4. Sampling the response function for each level of each factor.
5. Construction of partial dependencies of the response function on each factor.
6. Approximation of partial dependencies and derivation of a generalized mathematical model.

The main factors (input parameters) were determined: the content of the acrylic varnish in the aqueous solution (CAD, %: 0–30), and the concentration of surfactants (CS, g/dm^3^: 0–1). 

The numerical values of the levels for each factor are presented in Table 1.

A full-factor experiment was carried out on the basis of a constructed orthogonal, multi-level design matrix of a two-factor experiment (Table 2).

The response function was taken as the average particle diameter of the pigment (APDP, microns), the content of fine fractions (CF, %), and adsorption (A, g/g), which were determined experimentally.

After providing the active experiments according to Table 2, the experimental array was sampled for each level of each factor according to Table 3.

On the basis of a sample of the experimental dataset (Table 3), the partial dependences of the response function on the content of the film-forming agent and the concentration of the surfactant were constructed.

At the last stage, the partial dependences were approximated to obtain one-parameter equations characterizing the influence of each factor on the response function separately. To construct a multivariate statistical mathematical model (generalized equation) we used the formula proposed by M.M. Protodyakonov, which in the case of a two factorial experiment takes the following form (1):

(1) $$y=\frac{f\left(x_{1}\right)\cdot f\left(x_{2}\right)}{{\overset{–}{y}}^{m-1}}$$

where *f* (*x*_1_) and *f* (*x*_2_) are the partial dependences of the response function; $\bar{y}$ is the average value of the actual value of the output parameter for all n experiments (general average); and m is the number of factors. 

The $\bar{y}$ values were calculated using Formula (2):

(2) $$\overset{–}{y}=\frac{\sum_{i=1}^{n}y_{i}}{n}$$

where *y_i_* is an experimental value of the response function in experiment number *i*; and n is the total number of experiments in the matrix design.

The estimation of the accuracy of the obtained mathematical models was estimated using the coefficient of nonlinear multiple correlation (*R*) and significance (*t_R_*), which were calculated using Equations (3) and (4):

(3) $$R=\sqrt{1-\frac{\left(n-1\right)\cdot \sum_{i=1}^{n}{\left(y_{i}-{\overset{–}{y}}_{i}\right)}^{2}}{\left(n-p-1\right)\cdot \sum_{i=1}^{n}{\left(y_{i}-\overset{–}{y}\right)}^{2}}},$$

where *n* is the number of experiments; *p* is the number of input (independent) parameters; *i* is the serial number of the experiment; *y_i_* is the actual value of the output parameter in the *i* experiment; ${\hat{y}}_{i}$ is the calculated value of the output parameter, calculated using a multi-factor mathematical model, for the conditions (values of input parameters) of the *i* experiment; and $\bar{y}$ is the average value of the actual value of the output parameter for all *n* experiments (the general average).

(4) $$t_{R}=\frac{R\cdot \sqrt{n-p-1}}{1-R^{2}}$$

### 2.6. Methodology for Checking the Quality of Coatings

The corrosion rate was determined in 10% sulfuric acid solution (by weight) in accordance with the ISO 11845:2020 (en) standard for steel plates measuring 65 mm × 25 mm × 1 mm (length × width × thickness). The test time was 60 min. The corrosion rate as a function of time was calculated using the following equation: corrosion rate (g/(m^2^ × min)) = (m_1_–m_0_)/(S×t), where m_0_ and m_1_ represent the weights of the plates before and after immersion in sulfuric acid solution, respectively; S = 0.00325 m^2^ is the total area of the plate; and *t* is test time in minutes. The corrosion rate was determined by the gravimetric method (weighing plates before and after exposure in 10% sulfuric acid solution). The coating was applied to the plates by pouring and was kept for 24 h at a temperature of 20 ± 5 °C. The test time was 60 min. Each sample was weighed at least 5 times. The results show average values.

The adhesion of the coatings to the steel substrate was evaluated in accordance with the ISO 11845:2020 (en) standard for steel plates measuring 150 mm × 70 mm × 2 mm (length × width × thickness) in natural sunlight. The coating was applied to the plates by pouring and was kept for 24 h at a temperature of 20 ± 5 °C. All samples were examined at least 5 times. The results show average values. 

The appearance of the coatings was evaluated on steel plates measuring 150 mm × 70 mm × 2 mm (length × width × thickness) in natural sunlight. The coating was applied to the plates by pouring and was kept for 24 h at a temperature of 20 ± 5 °C. All samples were examined at least 5 times. The results show average values.

## 3. Results

### 3.1. Surface Active Properties of Surfactants at Interface Boundaries with Air and Solid Surface

**Figure 2:**
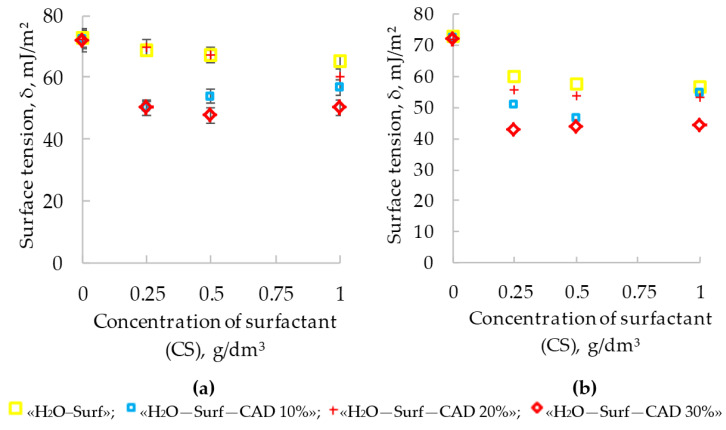
Surface tension isotherms (T = 298 K) in systems: (**a**) NaPA, (**b**) PC.

The presented surface tension isotherms (T = 298 °K) clearly reflected the dynamics of changes in σ at the water–air interface when adding surfactants to water solutions (Figure 2).

Both studied additives (NaPA and PC; Figure 2) lowered the surface tension both in pure water and in the presence of acrylic varnish (CAD 10, 20, and 30%). Therefore, NaPA and PC in this system should be considered as surfactants.

**Figure 3:**
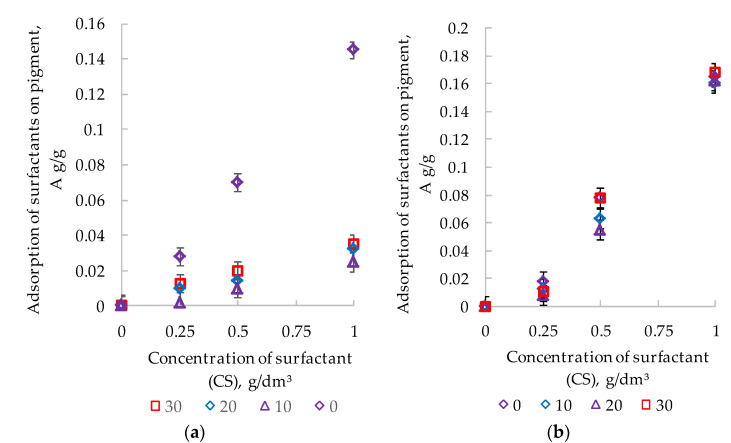
Isotherms (T = 298 K) of adsorption in the presence of PC (**a**) and NaPA (**b**) with the content of the film-forming agent in the system: 1—0%, 2—10%, 3—20%, 4—30%.

The equilibrium adsorption isotherms (T = 298°K) showed that with an increase in the additive concentration, the quantity of surfactants (Figure 3, curves 1a,b) on the interfacial surface of titanium dioxide with water increased.

The surface activity indicators were used to compare the ability of two surfactants to concentrate on the surface of the pigment and thereby reduce its specific surface energy.

The surface activity of surfactants (*dA/dCS*) was calculated from the slope tangent of the linear sections of the adsorption isotherms. The numerical coefficients of the linear equations describing the adsorption isotherms were determined using the least squares method. The values of the correlation coefficients were not lower than 0.95.

In water suspensions of TiO_2_, the surface activity of NaPA was 0.172 dm^3^/g; for PC, this indicator was slightly lower—0.147 dm^3^/g (Table 4).

### 3.2. Dispersing Effect of Additives in Water and Acrylic Suspensions of Titanium Dioxide

**Figure 4:**
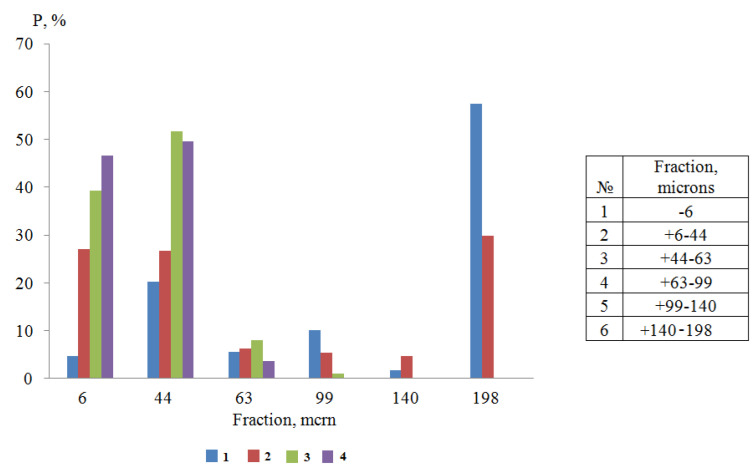
Distribution of titanium dioxide dispersions by size classes in suspensions containing a film-forming agent: 1—0%, 2—10%, 3—20%, 4—30%.

According to the results of computer-micro-optical analysis of water suspensions of titanium dioxide (CAD 0%), it was found that in the absence of surfactants, large fractions larger than 140 microns predominated (*P* = 57.5%). The content of fine fractions (particle size less than 6 microns) did not exceed 5% (Figure 4), and the average particle size was 13.12 microns.

**Figure 5:**
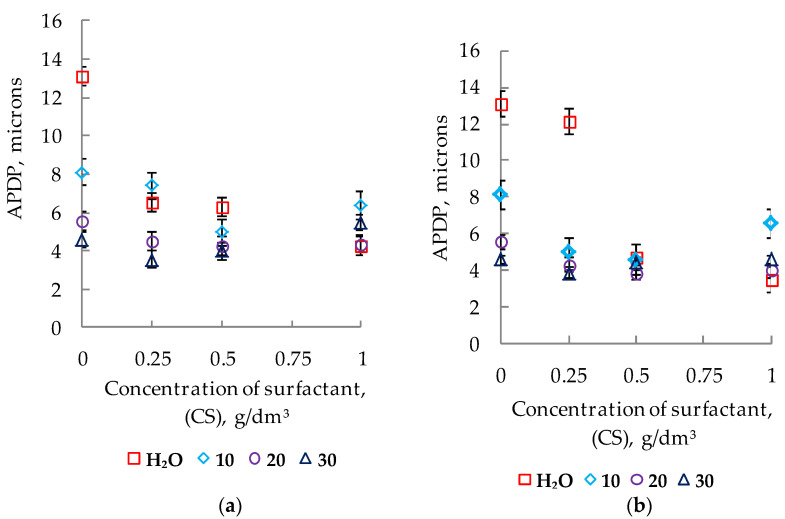
Change in the average particle diameter of the pigment in the presence of NaPA (**a**) and PC (**b**) with the content of the film-forming agent in the system.

The maximum disaggregating effect with respect to titanium dioxide, judging by the nature of the change in the average diameter and the CF, was recorded when dosing in a surfactant suspension at the level of 1 g/dm^3^ (Figure 5 curves 1a,b).

**Figure 6:**
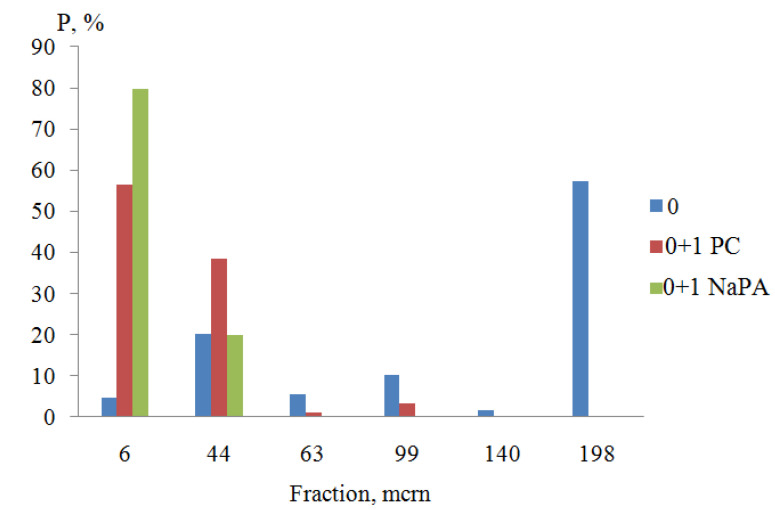
Distribution of titanium dioxide dispersions by size classes in suspensions with a concentration of surfactant CS = 1 g/dm^3^.

The presented diagram (Figure 6) shows that NaPA, without changing the content of fractions with a size of +6–44 microns, increased the number of small aggregates (≤6 microns) due to the absolute destruction of all other fractions (+63–198 microns).

**Figure 7:**
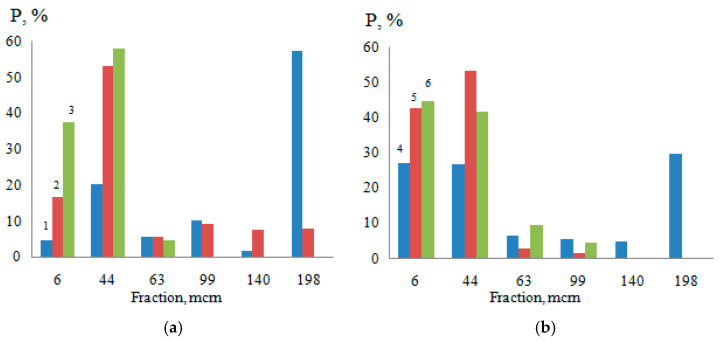
Distribution of titanium dioxide dispersions by size classes in water (**a**) and in a 10% film-forming solution (**b**) at CS = 0.5 g/dm^3^ (1—H_2_O, 2—H_2_O + 0.5 g/dm^3^ (PC), 3—H_2_O + 0.5 g/dm^3^ (NaPA), 4—10%, 5—10% + 0.5g/dm^3^ (PC), 6—10% + 0.5 g/dm^3^ (NaPA)).

According to the results of computer-micro-optical analysis of water–acrylic suspensions of titanium dioxide in the absence of surfactants, it was found that with 10% CAD (Figure 7), there was a partial destruction of large aggregates (99–198 microns), accompanied by an increase in the content of CF up to 27%. 

Experimental data showed that the nature of the change in the dispersed composition of TiO_2_ in acrylic suspensions was consistent with the individual contributions of the film-forming agent and surfactant. 

A comparative analysis of the graphs (Figure 7) shows that the introduction into the water of 0.5g/dm^3^ NaPA increased the content of fine fractions (CF) (≤6 µm) by 32.3%, and the units medium size (+6–63 µm) was 36.6%. This was due to the complete destruction of the large size fractions (+99–198 µm), which was in the absence of surfactants of 69.41%. For water-acrylic suspensions (CAD 10%), with the same content of NaPA, there was an increase in CF (≤6 microns) only by 17.6% and medium-sized aggregates (+6–63 microns) only by 17.9%, which was approximately 2.0 times less than water. This change in the dispersed composition of the pigment occurred as a result of the destruction of large aggregates ranging in size from 99 to 198 microns, which were only 39.8% in the water suspension of the acrylic varnish, i.e., 1.7 times less than in water. As a result, in this suspension, the minimum APDP (4.6 microns min) was greater than the same value in water (3.5 microns min).

PC, at the same concentration (CS = 0.5 g/dm^3^) in the water–acrylic suspension, destroyed primarily the largest aggregates (140 microns) and partially fractions of size +63–99 microns, which was accompanied by the appearance of more particles of size +6–44 microns than small ones (≤6 microns), in comparison with NaPA. As a result, the minimum APDP in a water–acrylic suspension with 10% CAD and 0.5 g/dm^3^ of PC (APDP min 4.9 microns) did not have such a big difference with the wedging effect in water (APDP min 4.3 microns).

Thus, surface-active additives, when they are dosed into water–acrylic compositions, destroy aggregates much smaller in size than they do in water suspension.

**Figure 8:**
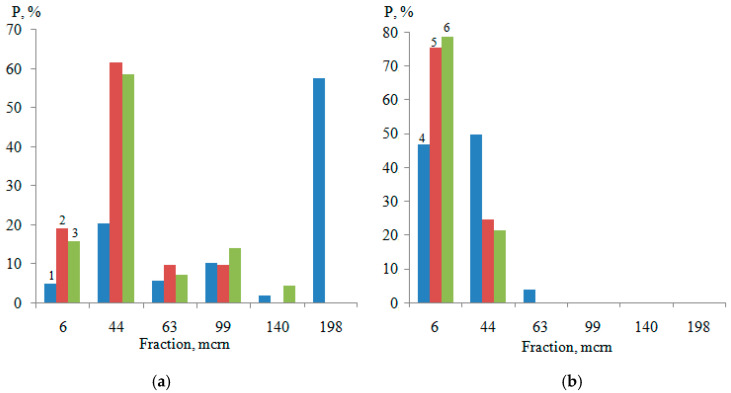
Distributions of titanium dioxide dispersions by size classes in water (**a**) and in a 30% film-forming agent solution (**b**) with CS = 0.25g/dm^3^ (1—H_2_O, 2—H_2_O + 0.25g/dm^3^ (PC), 3—H_2_O + 0.25g/dm^3^ (NaPA), 4—30%, 5—30% + 0.25g/dm^3^ (PC), 6—30% + 0.25g/dm^3^ (NaPA)).

Distributions of titanium dioxide dispersions by size classes in water and in a 30% film-forming agent solution with CS = 0.25g/dm^3^ are presented in Figure 8.

**Figure 9:**
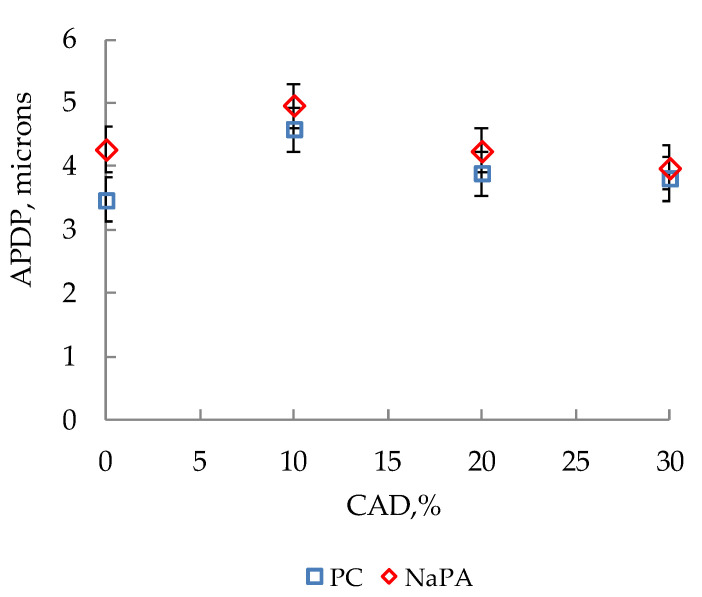
Changes in the minimum average diameter of particles in suspensions with different film-forming agent content.

Changes in the minimum average diameter of particles in suspensions with different film-forming agent content are presented in Figure 9.

### 3.3. Optimization of Compositions of Paints

The results of an active experiment aimed at deriving a mathematical model of the process of dispersing titanium dioxide in an acrylic film-forming agent are presented in Table 5.

A sample of the values of the APDP and the CF by the levels of the CS and CAD presented in Table 6.

Partial dependencies were approximated by one-parameter equations, which were combined into multivariate mathematical models (Equations (5–8)).

System “Acrylic film-forming agent—PC—TiO_2_”

(5) $$APDP=\frac{\left(6.029\cdot CS^{2}-8.136\cdot CS+7.204\right)\cdot \left(-0.5\cdot CAD^{0.58}+7.8\right)}{5.62},\;\mathrm{microns},$$

(6) $$CF=\frac{\left(-67.79\cdot CS^{2}+83.52\cdot CS+28.9\right)\cdot \left(3.2\cdot CAD^{0.74}+23\right)}{44.14},\;\%.\;$$

System “Acrylic film-forming agent—NaPA—TiO_2_”

(7) $$APDP=\frac{\left(6.38\cdot CS^{2}-9.753\cdot CS+7.983\right)\cdot \left(-0.93\cdot CAD^{0.43}+8.37\right)}{5.82},\;\mathrm{microns},\;$$

(8) $$CF=\frac{\left(26\cdot CS^{0.39}+29\right)\cdot \left(\left(7.3\cdot 10^{-4}\cdot CAD^{4.58}\right)\cdot {\left(18.2+{\left(CAD-15.9\right)}^{2}\right)}^{-1}+34\right)}{43.9},\;\%.\;$$

The calculations showed satisfactory convergence of the experimental and calculated response function values (for the 95th level of significance): R> 0.92 and t_R_ > 2.

### 3.4. The Effect of Surfactants on the Quality of Coatings

**Figure 10:**
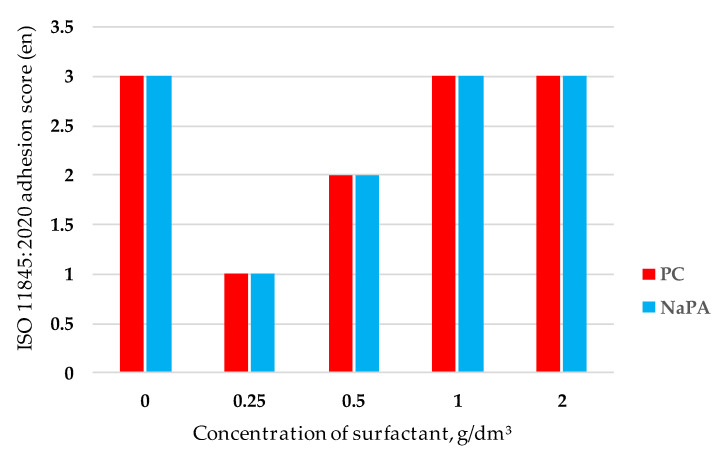
The effect of surfactants on the adhesion of the coating of the studied paints.

The effect of surfactants on coating adhesion is shown in Figure 10.

**Figure 11:**
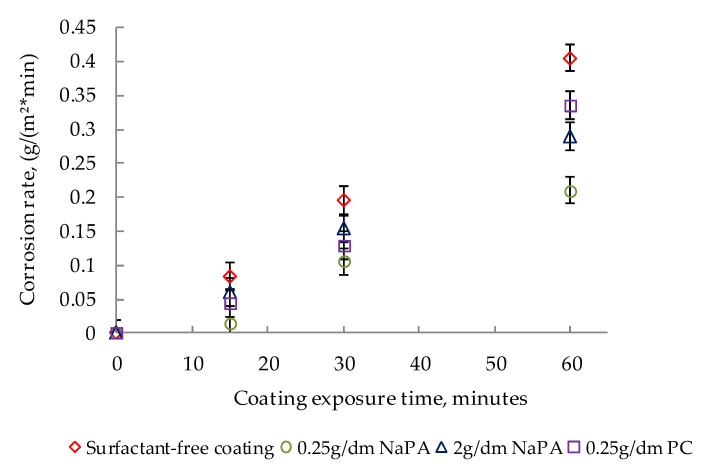
The effect of surfactants on the corrosion rate of the studied paints in 10% sulfuric acid solution.

The effect of surfactants on the corrosion rate in a 10% sulfuric acid solution is shown in Figure 11.

**Figure 12:**
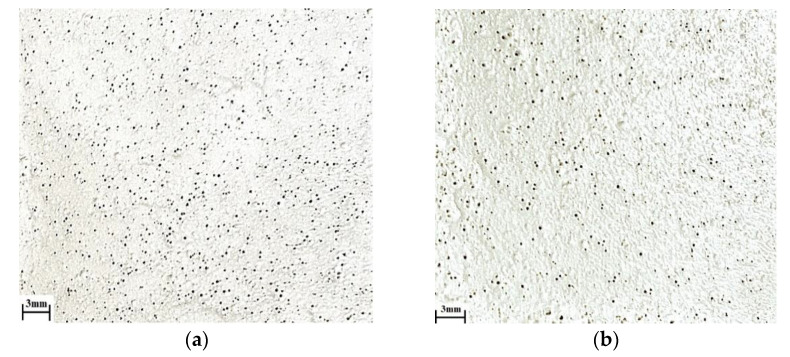
The effect of surfactant content on the appearance of the coating: (**a**) without surfactants; and (**b**) with NaPA 0.25g/dm^3^.

The effect of surfactants on the appearance of the coating is shown in Figure 12.

## 4. Discussion

Comparative analysis of the specific surface energy values (σ, mJ/m^2^) at the interface with air shows that NaPA exhibits the highest surface activity in water (Figure 2, curve 1a). In the range of NaPA concentrations from 0 to 1 g/dm^3^, the surface tension (σ) decreased by 15.32 mJ/m^2^ and amounted to 56.72 mJ/m^2^. For the PC at the same concentration site (Figure 2, curve 1b), in comparison with the NaPA, the surface tension depression was almost 2.00 times lower (Δσ = 7.64 mJ/m^2^). The appearance of a more energetically favorable interfacial surface of titanium dioxide was accompanied by a decrease in the concentration of surfactants at the boundary with air and, as a result, an increase in the value of σ (Figure 2 curve 2a) at 2.26 mJ/m^2^ (NaPA) and at 3.43 mJ/m^2^ (PC), at CS = 1.00 g/dm^3^ (Figure 2, curve 2b).

The adsorption rates are maximum when the surfactant is dosed into a water suspension of 1 g/dm^3^. For water–acrylic suspensions of titanium dioxide, a decrease in the adsorption of PC was observed in comparison with those of water (Figure 3, curves 2–4a). In compositions with CAD 10%, the surface activity index of PC is 0.026 dm^3^/g (Table 4), which is 5.7 times lower than in the solvent *(dA/dCS = 0.147 dm^3^/g).* However, with a further increase in the content of the CAD (20 and 30%), the decrease in the surface activity of the PC slows down. At CAD 30%, the surface activity on the pigment is reduced to 0.040 dm^3^/g, i.e., only 3.7 times compared to water. In contrast to PC, when NaPA is introduced into acrylic-containing compositions (Figure, 3 curves 2–4b), the adsorption values are found to be close to those in water (Figure 3, curve 1b). In the isoconcentrated suspensions with the content of NaPA and film-forming agent suspensions, the surface activity coefficient of *dA/dCS* remained at the level of 0.167–0.178 dm^3^/g (Table 4). This clearly indicates the absence of competitive adsorption between the polymer and the introduced surfactant, i.e., the NaPA is sorbed on the active sites of the pigment, free of the film-forming macromolecules. As a result, the difference between *dA/dCS* and the two surfactants in water–acrylic suspensions is aggravated and becomes very significant. The surface activity of NaPA exceeds PC from 6.4 to 4.5 times as the CAD increases from 10 to 30%. The ability of NaPA and PC to adsorb on the surface of the pigment and thereby lower the value of σ at the interface suggests their dispersing activity in accordance with the Rebinder classification [17].

The introduction of both the surfactant polyether siloxane copolymer and sodium polyacrylate into the compositions opens up additional possibilities for purposeful changes in the dispersed composition, which became the subject of our further research. When adding additives to water suspensions, significant changes in the characteristics of the dispersed composition of the pigment were noted (Figure 5, curves 1a and 1b; Table 4). The degree of disaggregation of titanium dioxide was used as a measure to assess the disjoining effect of surfactants. The minimum values of the APDP and the maximum of CF (%) correspond to the maximum of the surfactant disjoining activity.

With a change in the concentration of NaPA in water from 0 to 1 g/dm^3^, the APDP decreased by 3.8 times and amounted to 3.5 microns (Figure 5 curve 1a), and the content of fine (≤6 microns) fractions increased by 17.0 times and reached 80.0% (Figure 6). As a result of the adsorption-disjoining action of PC at the same additive concentration (CS = 1 g/dm^3^), the particle size decreased only to 4.3 microns (Figure 5, curve 1b), and the content of fine fractions increased to 56.7% (Figure 6). In contrast to NaPA, under the influence of PC, large fractions were destroyed not only to small fractions (≤ 6 microns), but also to medium-sized aggregates (−44 + 63 microns).

Thus, NaPA, which provides a greater (1.22 times) decrease in the interfacial surface energy at the interface of the pigment with water (Figure 3, curves 1a and 1b), shows a greater (1.17 times) increase in the dispersion of titanium dioxide in comparison with PC. With a further increase in the content of the film-forming agent (20–30%), the number of small fractions increases to 39 and 47%, respectively, while the complete destruction of large aggregates with a size of 99 to 198 microns occurs. At this stage, first of all, the particles connected mainly by point contacts are disaggregated, and only then by stronger linear and planar contacts [18].

The introduction of surfactants into the compositions opens up additional possibilities for purposeful changes in the dispersed composition. The depth and direction of these changes under the influence of surfactants in systems with a film-forming agent differs from water suspensions.

According to experimental data, the introduction of two types of surfactants into water–acrylic suspensions leads to a decrease in the size of TiO_2_ particles in a limited concentration range (Figure 5, curves 2–4a and 2–4b).

The maximum disaggregating activity of surfactants in water–acrylic suspensions (CAD = 10–20%) was recorded at their concentration at 0.5 g/dm^3^ (Figure 5, curves 2–3a,b). In more concentrated suspensions (CAD = 30%), the best characteristics of the splitting and disaggregation of pigment particles by additives are provided in the region of lower (CS = 0.25 g/dm^3^) concentrations (Figure 5, curves 4a,b).

Outside the specified surfactant concentration areas, the TiO_2_ particles were enlarged. Aggregation processes are significantly intensified in suspensions with 10 and 30% content of the film-forming agent. In suspensions (CAD = 10%), these processes are most pronounced in the presence of NaPA. Thus, with an increase in the concentration of NaPA from 0.5 to 1 g/dm^3^, the average particle diameter of the pigment increased by 1.99 microns (from 4.58 to 6.57 microns). For the PC, at the same concentration site, the increase in the size of the dispersions was less significant—by 1.68 microns (from 4.95 to 6.63 microns). In more concentrated (CAD = 30%) suspensions, with excessive surfactant dosing (0.25 g/dm^3^), the effect of secondary aggregation for PC remains approximately at the same level (ΔAPDP = 1.94 microns); particle enlargement from 3.56 microns to 5.50 microns with an increase in PC from 0.25 to 1 g/dm^3^. For NaPA, the increase in the average diameter from 3.80 microns to 4.60 was significantly smaller (ΔAPDP = 0.80 microns) compared to the suspension (CAD = 10%).

At the same time, the higher the content of the film-forming agent in the suspensions, the lower the contribution of surfactants to the disaggregation of titanium dioxide dispersions. Thus, at CAD = 10% and CS = 0.5 g/dm^3^, the APDP value decreased relative to systems without surfactants by 3.15 microns (PC) and by 3.51 microns (NaPA), and in suspensions with CAD = 30% and a similar surfactant content, this difference was 0.62 microns (PC) and 0.106 microns (NaPA).

This is primarily due to the change in the size distribution of titanium dioxide dispersions under the influence of the film-forming agent in water–acrylic suspensions, in comparison with water (Figure 7). The results of computer-micro-optical analysis of compositions with 30% content of film-forming agent and 0.25 g/dm^3^ surfactants show (Figure 8b) that in the absence of large aggregates (+99–198 microns), the adsorption-disjoining action of both additives increases the content of particles with a size +6–44 microns only by 3.74%, due to the destruction of fractions of +44–63 microns. As a consequence, the minimum average particle diameter of pigment in the presence of NaPA (APDP_min_ = 3.80 microns) is close to the same indicator with PC (APDP_min_ = 3.56 microns).

Moreover, the smaller the aggregate in size, the fewer defects (cracks, cavities, etc.) in the articulation of the particles, and the more difficult the surfactant penetrates into their cavities and the more difficult it is to destroy them. As a result, the depth of dispersed changes in the pigment under the influence of film-forming agent content in acrylic suspensions decreases in comparison with water (Figure 9). These results allow us to understand that the distribution of solid-phase particles by size in a liquid dispersion medium has a decisive influence on the processes of destruction of pigment aggregates under the influence of surfactants. A comparison of the dispersing activity of two surfactants, with the same particle size distribution of the dispersed phase, demonstrates the second determining role in the processes of pigment disaggregation. This role consists of reducing the interfacial surface energy at the interface of the pigment with the liquid medium by the additives.

Computer-micro-optical analysis showed that the introduction of the same surfactant concentration (CS = 0.5 g/dm^3^) into the same suspension (CAD = 10%) causes the maximum intensification of the process of dispersion of pigment particles. However, the effect of intensification under the influence of NaPA and PC is different. A comparative analysis of the surfactant adsorption parameters (Figure 3) shows the reason for these differences. The adsorption value of NaPA is 0.03 g/g, which is three times higher than the ability of PC to accumulate on the surface of the pigment (A = 0.01 g/g). As a result, NaPA, which provides a greater adsorption reduction in strength, shows a greater dispersing effect than PC. The maximum disaggregation effect for NaPA and PC at the same surfactant concentration (CS = 0.25 g/dm^3^) in a suspension with a high film-forming agent content (CAD = 30%) has an equal dispersing effect.

This is due to the proximity of the adsorption values of NaPA (0.012 g/g) and PC (0.010 g/g) in these suspensions. The same reduction of the interfacial surface energy by the additives at the interface of the pigment with the bulk phase creates a similar effect of the splitting action of surfactants. 

**Figure 13:**
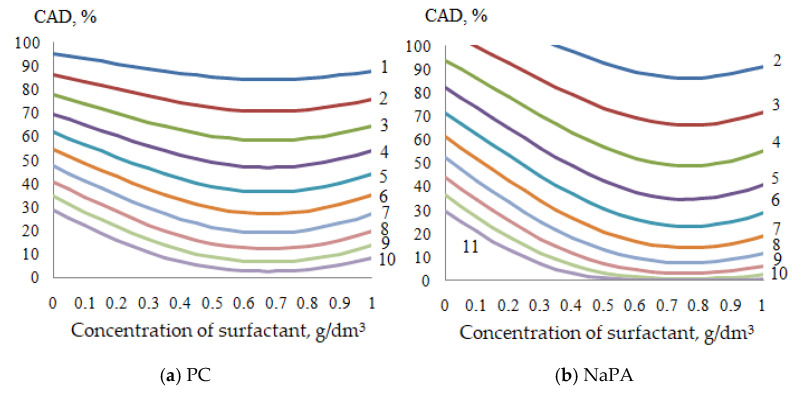
Lines of fixed average particle diameter of pigment depending on the content of the film-forming agent and the concentration of surfactants. APDP, microns: 1—1.0; 2—1.5; 3—2.0; 4—2.5; 5—3.0; 6—3.5; 7—4.0; 8—4.5; 9—5.0; 10—5.5; 11—6.0.

On the basis of generalized Equations (5) and (7), nomograms are obtained (Figure 13), which allow the values of the above parameters to be determined to achieve fixed values of the average diameter (APDP). Thus, the values of APDP = 4 microns with a film-forming agent content of 30% in the system can be achieved at a concentration of NaPA 0.29 g/dm^3^ and PC 0.35 g/dm^3^. With an increase in the film-forming agent content (40%), the same particle size can be achieved with a lower concentration of NaPA (CS = 0.12 g/dm^3^) and PC (CS = 0.23 g/dm^3^). Thus, two-factor nomograms are constructed that allow applied problems to be solved, in particular, optimizing the compositions of acrylic dispersions.

The introduction of surfactants (Figure 10) into paints improves the adhesion of the coating (from 3 points to 1 point according to ISO 11845:2020 (en)). Both investigated surfactants improve adhesion equally. Moreover, the optimum of dispersing activity of surfactants corresponds to the maximum adhesion of the coating.

The introduction of surfactants (Figure 11) into paints reduces the rate of acid corrosion. With the NaPA content at a concentration of 0.25 g/dm^3^, the corrosion rate decreased by half (from 0.405 to 0.21 g/m^2^ × min). A similar decrease in the corrosion rate is typical for PC.

The introduction of surfactants (Figure 10) into paints improves the adhesion of the coating (from 3 to 1 point according to ISO 11845:2020 (en)). Both investigated surfactants improve adhesion equally. Moreover, the optimum of dispersing activity of surfactants corresponds to the maximum adhesion of the coating.

The introduction of surfactants (Figure 11) into paints reduces the rate of acid corrosion. With the NaPA content at a concentration of 0.25 g/dm^3^, the corrosion rate decreased by half (from 0.405 to 0.21 g/m^2^ × min). A similar decrease in the corrosion rate is typical for PC. 

According to Figure 12, the coating formed in the presence of NaPA contains significantly fewer pores than the coating without surfactants. 

The logical continuation of this study is to further study the effect of the additives in question on the physico-mechanical (hardness, impact resistance, etc.) and decorative (gloss, color, etc.) properties of coatings. It would be especially interesting to check the effect of surfactants on the anticorrosive properties of wooden objects, since acrylic paints are often used to paint wooden surfaces.

## 5. Conclusions

(1) According to the results of the conducted studies, the possibility of using polyether siloxane copolymer and sodium polyacrylate in paint and varnish compositions based on an aqueous dispersion of acrylic varnish and titanium dioxide as modifying additives of dispersing action is proved.
(2) A narrow range of concentrations of two types of surfactants providing the maximum characteristics of splitting and disaggregation of pigment particles is revealed. A minimum of the average diameter of the pigment with a film-forming agent content of 10 and 20% is provided with the introduction of 0.5 g/dm^3^. In more concentrated suspensions of CAD = 30%, it is required to reduce the introduction of surfactants by 2 times (CS = 0.25 g/dm^3^). Excessive concentration of surfactants leads to secondary aggregation processes.
(3) It was found that the larger the size of the aggregates of the pigment particles, the greater the disjoining pressure created by the surfactant. The depth of dispersal changes in the pigment under the influence of surfactants in acrylic suspensions decreases, in comparison with water, as a result of the destruction of large aggregates by the film-forming agent.
(4) With the same distribution of pigment particles by size, the effect of surfactants is higher, and the greater the value of the adsorption-disjoining action. Sodium polyacrylate, which provides a greater adsorption reduction in strength, shows a greater dispersing effect than polyether siloxane copolymer. The adsorption value of NaPA is 0.03 g/g, which is three times higher than the ability of PC to accumulate on the surface of the pigment (A = 0.01 g/g) with the same dosage of surfactants (CS = 0.5 g/dm^3^) into the suspension (CAD = 10%).
(5) The maximum disaggregation effect for NaPA and PC at the same surfactant concentration (CS = 0.25 g/dm^3^) in a suspension with a high film-forming content (CAD = 30%) has an equal dispersing effect as a result of the proximity of the adsorption parameters of NaPA (0.012 g/g) and PC (0.010 g/g).
(6) A multivariate equations was obtained that generalizes the combined contribution of surfactant concentrations and the content of the film-forming agent in suspensions to the APDP and CF.
(7) The introduction of surfactants in the composition with a content of 0.25 g/dm^3^ allows the rate of acid corrosion to be reduced by two times and to significantly increase the adhesion of the coating (by two points according to ISO 11845: 2020).

## Figures and Tables

**Table 1:** Numerical values of levels for each factor.

The Factors	Level			
	1	2	3	4
CS, g/dm^3^ (x_1_)	0	0.25	0.5	1.0
CAD, % (x_2_)	0	10	20	30

**Table 2:** General view of a multilevel design matrix of a two-factor experiment.

x_1_Factor Levels	x_2_ Factor Levels			
	1	2	3	4
1	y_1_	y_5_	y_9_	y_13_
2	y_2_	y_6_	y_10_	y_14_
3	y_3_	y_7_	y_11_	y_15_
4	y_4_	y_8_	y_12_	y_16_

**Table 3:** Experiment factors levels and their corresponding samples.

Factor x_1_ Levels	Sample	Factor x_2_ Levels	Sample
CS, g/dm^3^		CAD, %	
0	(y_1_ + y_5_ + y_9_+ y_13_)/4	0	(y_1_ + y_2_ + y_3_ + y_4_)/4
0.25	(y_2_ + y_6_+ y_10_+ y_14_)/4	10	(y_5_ + y_6_ + y_7_ + y_8_)/4
0.50	(y_3_ + y_7_ + y_11_+ y_15_)/4	20	(y_9_ + y_10_ + y_11_ + y_12_)/4
1.00	(y_4_ + y_8_ + y_12_ + y_16_)/4	30	(y_13_ + y_14_ + y_15_ + y_16_)/4

**Table 4:** Indicators of surface activity of PC and NaPA at the interface with titanium dioxide.

CAD, %	PC		NaPA	
	*dA/dCS*, dm^3^/g	R^2^	*dA/dCS*, dm^3^/g	R^2^
0	0.147	0.996	0.172	0.978
10	0.026	0.963	0.167	0.966
20	0.031	0.989	0.170	0.948
30	0.040	0.984	0.178	0.964

**Table 5:** Multilevel design matrix of two-factor experiment.

CS, g/dm^3^	CAD, %							
	0		10		20		30	
	CF,%	APDP, Microns	CF,%	APDP, Microns	CF,%	APDP, Microns	CF,%	APDP, Microns
	PC							
1	56.73	4.261	23.618	6.362	61.043	4.295	36.458	5.498
0.5	16.59	6.303	42.674	4.945	63.136	4.244	96.637	3.978
0.25	19.11	6.541	24.362	7.393	57.803	4.498	75.373	3.562
0	4.712	13.121	27.141	8.093	39.197	5.554	46.652	4.593
	NaPA							
1	79.991	3.47	22.737	6.573	59.309	3.991	51.096	4.596
0.5	37.496	4.711	44.715	4.58	69.769	3.885	41.999	4.487
0.25	15.796	12.173	38.919	5.002	44.215	4.318	78.659	3.804
0	4.712	13.121	27.141	8.093	39.197	5.554	46.652	4.593

**Table 6:** Sample of response functions for each level of factors CS and CAD.

CS, g/dm^3^	CF, %	APDP, Microns	CAD, %	CF, %	APDP, Microns
	PC				
1	44.46	5.104	0	24.29	7.557
0.5	54.6	4.868	10	29.45	6.698
0.25	44.16	5.499	20	55.29	4.648
0	29.43	7.840	30	63.78	4.408
	NaPA				
1	53.28	4.658	0	34.50	8.369
0.5	48.49	4.416	10	33.38	6.062
0.25	44.40	6.324	20	53.12	4.437
0	29.43	7.840	30	54.60	4.370

## Data Availability

The datasets generated during and/or analyzed during the current study are available from the corresponding author upon reasonable request.

## Supplementary Materials

The following are available online at: https://www.mdpi.com/article/10.3390/polym14050996/s1, The Computer-Micro-Optical Method of Analysis (description of the method).
