# Supplementary material for: Exploration of the Adsorption Reduction of the Pigment Aggregates Strength under the Effect of Surfactants in Water-Dispersion Paints
Source: Polymers (Basel). 2022 Feb 28;14(5):996. doi: 10.3390/polym14050996 (PMC8912600; doi:10.3390/polym14050996)
Supplement: Supplementary file 1 [file polymers-14-00996-s001.zip › polymers-1536134-supplementary.pdf]

## THE COMPUTER-MICRO-OPTICAL METHOD OF ANALYSIS

The computer-optical method of analysis is based on the rational combination of the functional capabilities of optical analyzers and computer systems through the use of electronic converters equipped with a standard USB port, small-format CCD camera and software.

We used a computer-microscopic installation consisting of a microscope (CARL ZEISS 451422) connected to a personal computer using a small-sized color video nozzle HB-35, equipped with a standard USB port. The electronic video attachment is inserted into the upper tube of the microscope eye piece. The latter is equipped with removable lenses, which allows you to vary the magnification factor from 60 to 1000 units. The adjustment of the video image in terms of sharpness, contrast, and coverage area of the analyzed space was carried. Photofixing and image saving were performed by the Microsoft VIDCAP32 program, which demonstrates the main functionality of VideoForWindows (VFW) (can play video and work with video equipment) including the HB-35 video attachment. To get an image on the monitor in the Microsoft VIDCAP32 program, select the "Preview" option. We set the output and image resolution to 640x480 constant. The image was saved in the menu File, save frame as, setting the image name and file format "bmp".

The subsequent processing of video images within the framework of the algorithm used by us was carried out using a specialized program "Spectrum of differential distribution" (Figure S1). This program is an integral part of computer-micro-optical systems and allows you to process images in the "bmp" format to obtain quantitative information about the specific number of particles (per unit area), their geometric parameters (linear dimensions, configuration, area) and, finally, in general, about the fractional composition of emulsions, suspensions and powdery materials.

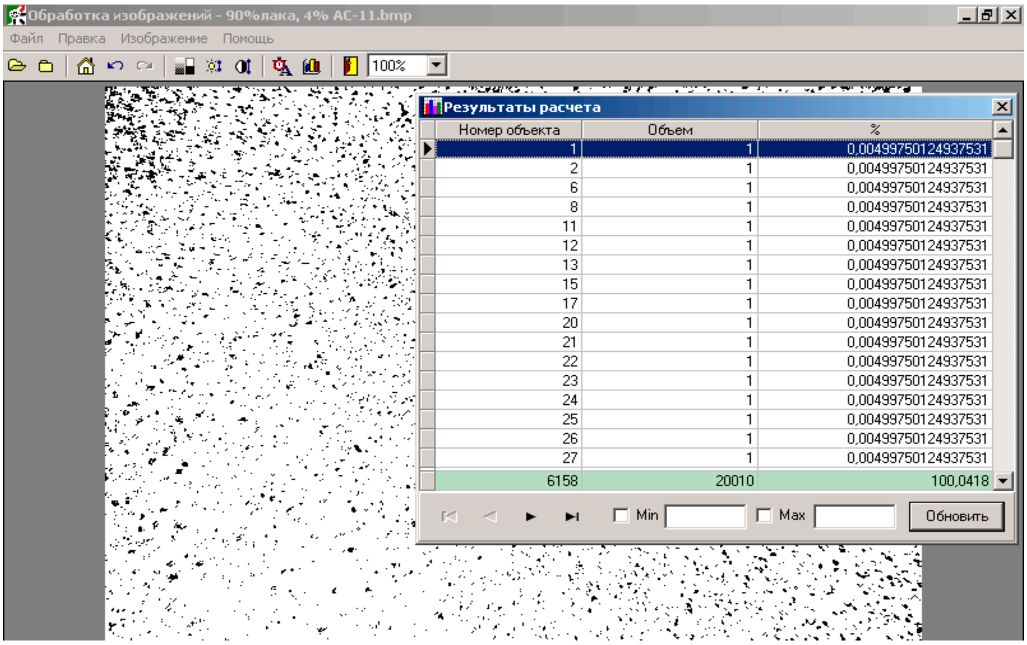


**Figure S1.** Image processing in the program "Spectrum of differential distribution"

The previously saved image was opened in the program "Spectrum of differential distribution":

- the program performed "binarization" (Figure S2) of the processed object. Binarization is the transformation of an image, in general, to a single color (most often to black and white). In Photoshop terms, this is called "at the 50% level", since it selects a certain threshold, all values below which turn into the background color (white), and above-into the main color (black);

Image processing using the "binarization" function

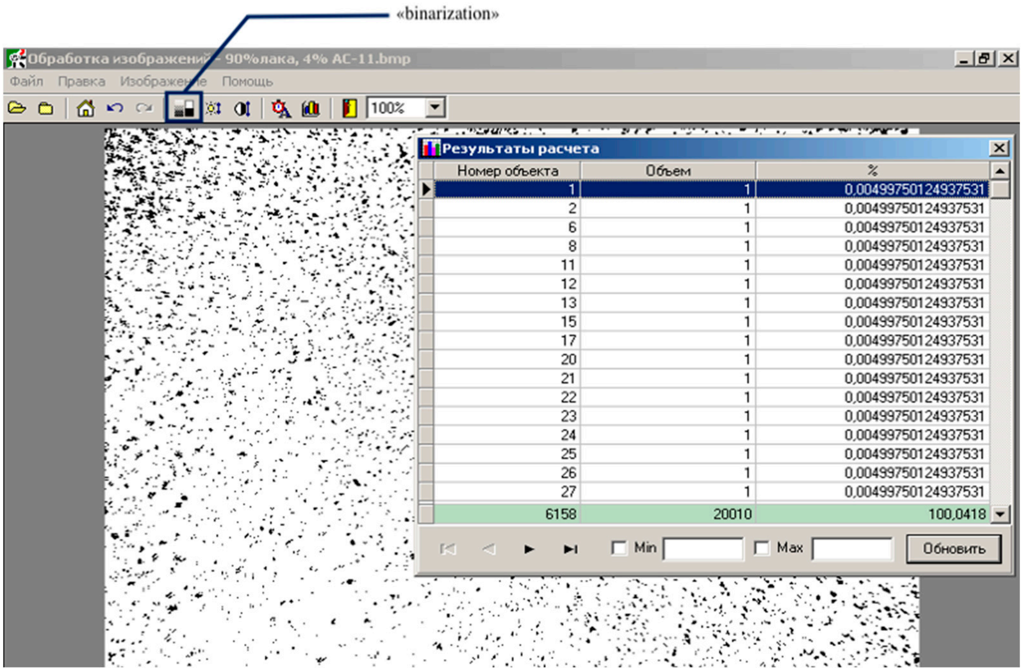


**Figure S2.** Image processing using the "binarization" function

- to calculate the number of objects, select the "Calculation" function on the toolbar (Figure S3);

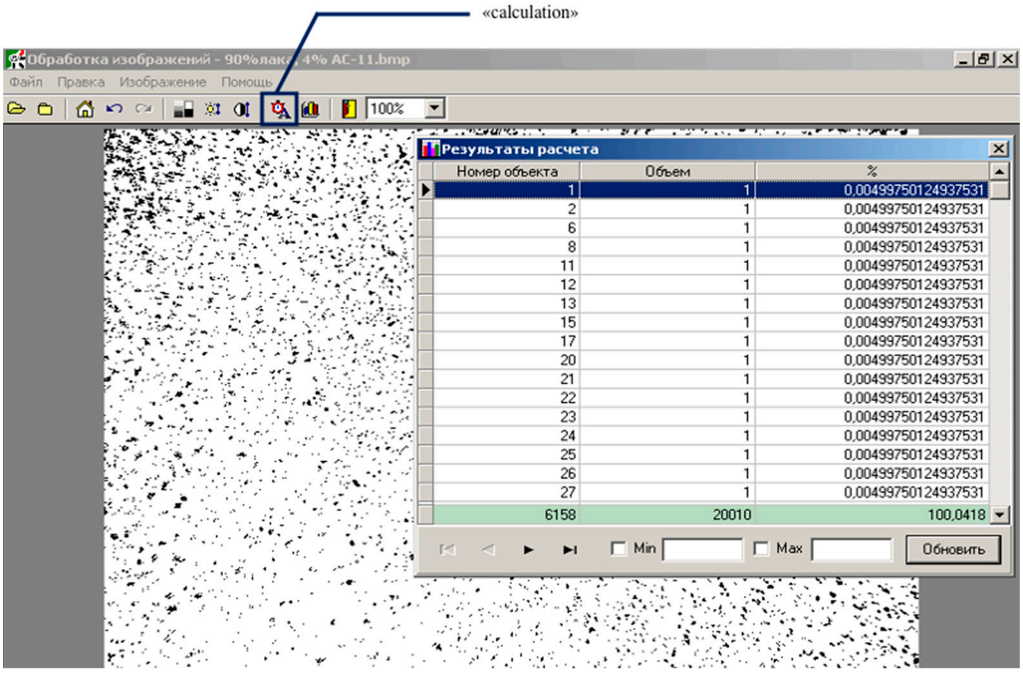


**Figure S3.** Image processing by the "Calculation" function

- next, we used the "Calculation Results" function (Figure S4).

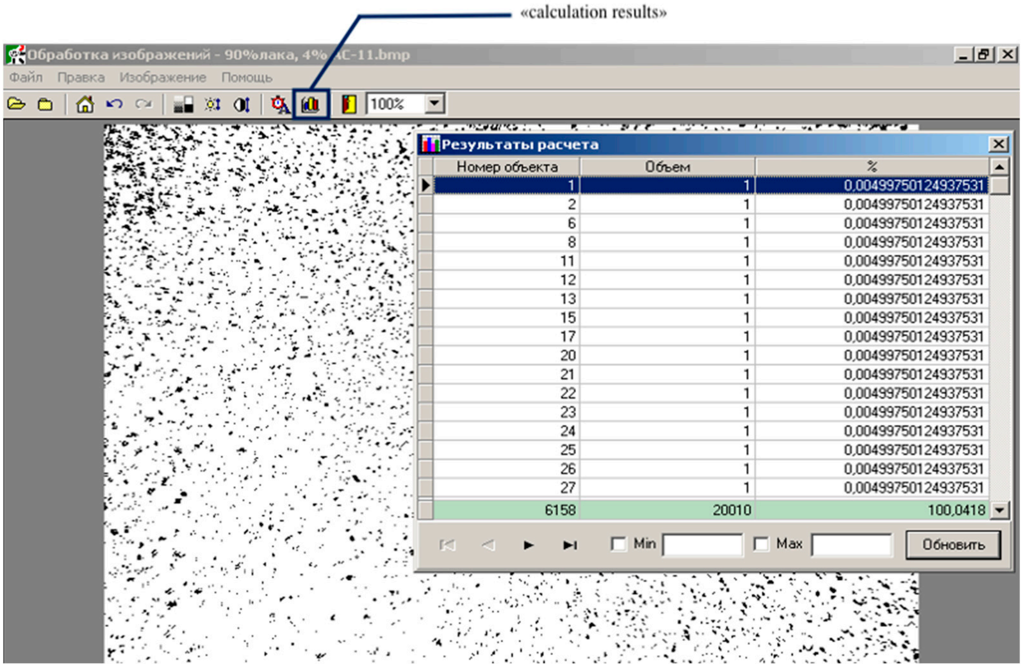


**Figure S4.** Function "Calculation results”.

After the last operation, a table is displayed on the screen, each row of which shows the characteristics of particles (objects): the sequence number in the image, the area they occupy in pixels, and their content in the image (Figure S5).

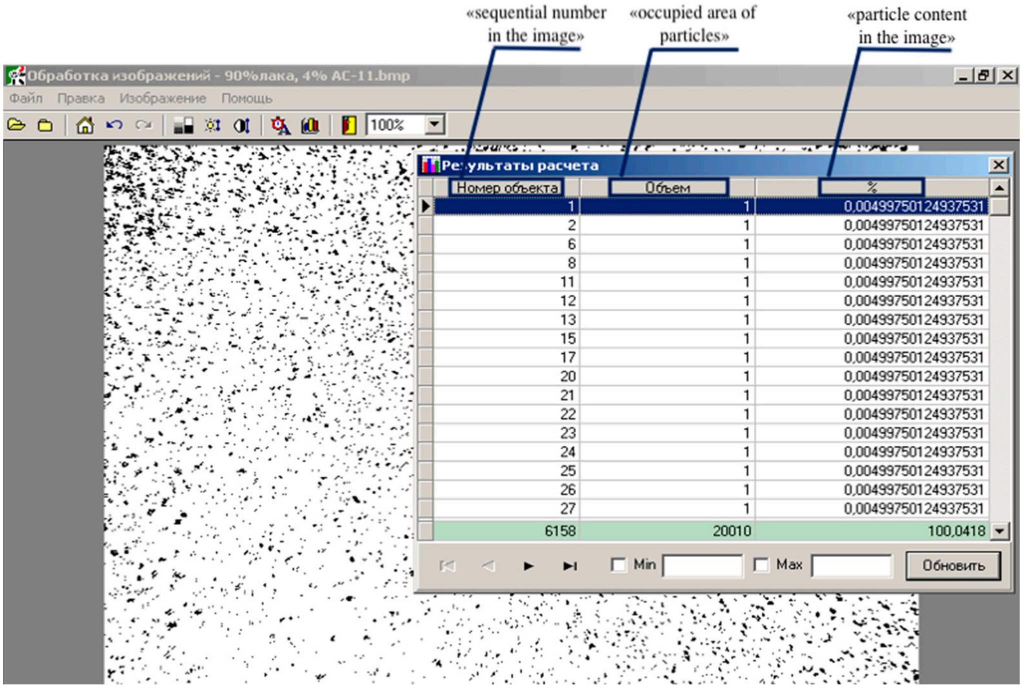


**Figure S5.** Characteristics of particles (objects): the sequence number in the image, the area they occupy in pixels, and their content in the image.

The last row of the table (highlighted in green) indicates the total number of objects (particles) in the image, the total area of all objects (pixel), the total particle content in the image which should ideally be equal to 100% (Figure S6).

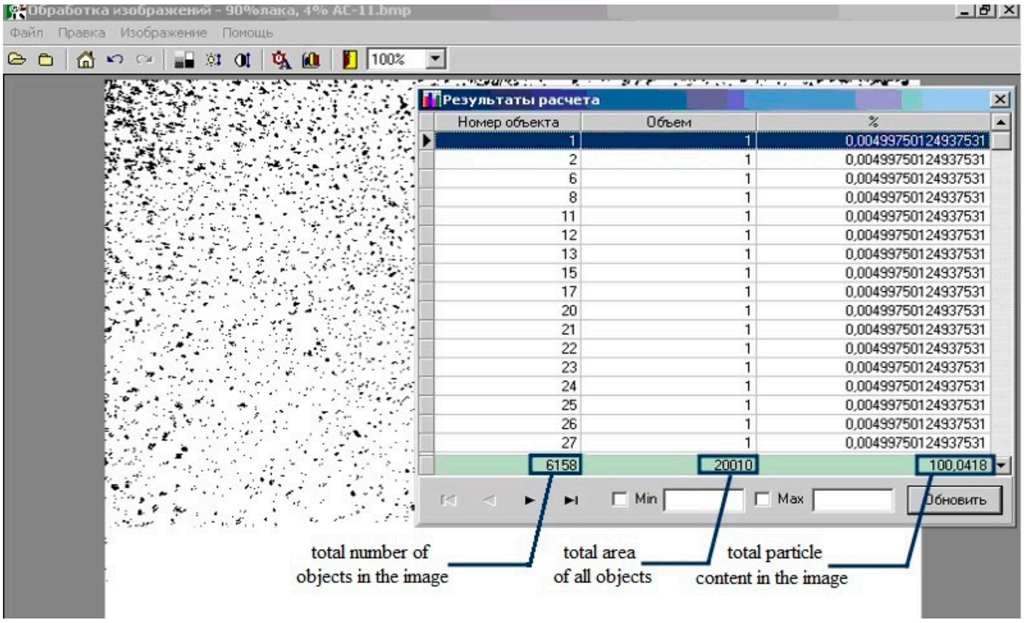


**Figure S6.** Functions: "total number of objects in the image", "total area of all objects" (pixel), and "total particle content in the image" (%).

Getting quantitative information about individual fractions is done by specifying the range of object sizes on the table panel (min, max). The program has a number of additional features that allow you to further adjust the brightness and contrast of failed images to get reproducible results when processing them. The measurement error of the program is ±0.05%.

It should be noted that in the current practice of finding the geometric parameters of particles, their size is taken to be the equivalent diameter or radius of a circle that has the same cross-section as irregularly shaped particles. The program "Spectrum of differential distribution" (used in computer-microscopic complexes) allows you to find the areas of particles expressed in pixels. A pixel is an indivisible rectangular object characterized by a certain color.

Finding the conversion coefficients into metric units for a given multiplicity of magnification of the microscopic installation consisted in finding the number of pixels of an object with a known metric area.

The object used was the small square of the Goryaev chamber, which is a thick rectangular transparent glass with two grids engraved on its surface (Figure S7). The grid of the Goryaev chamber is formed by a system of dividing lines drawn mutually perpendicular. It has 3600 small squares: side 1/20 mm, area 0.0025 mm$^2$, volume 1/4000 mm$^3$; 225 large squares: side 1/5 mm, area 0.04 mm$^2$, volume 1/250 mm$^3$. The side of the entire mesh is 3 mm, the area is 9 mm$^2$, the volume is 0.9 mm$^3$; the height of the chamber formed when grinding the ground cover glass is 0.1 mm.

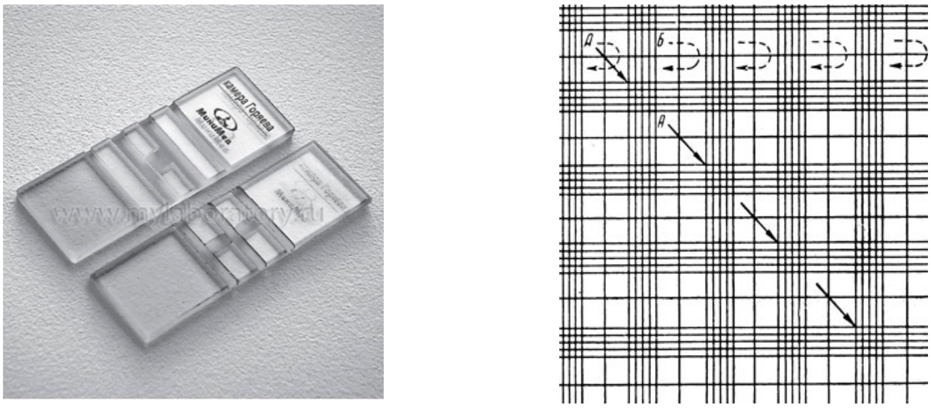

General view of the chamber    Goryaev chamber grid

**Figure S7.** Goryaev chamber

The images of the Goryaev chamber grid obtained at three magnifications of the microscopic system (x110, x350, x700) and saved using the Microsoft VIDCAP32 program were processed in the "Paint" program. An object (a small square) with a known area of 0.0025 mm$^2$ was cut out of the camera grid image. The contour of the cut square included the left and upper dividing lines and two sides along the contour of the grid square (Figure S8). The square was painted in black.

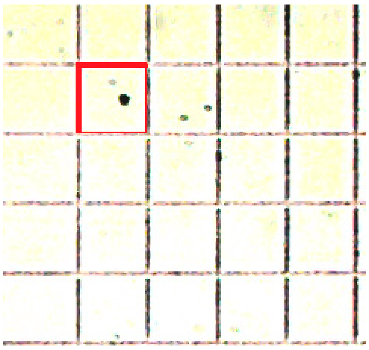

**Figure S8.** Forming an object with a given area of 0.0025 mm$^2$

The image of the squares cut from the Goryaev chamber grid at different magnifications of the microscopic system is presented in Table S1.

**Table S1.** Image of the Goryaev chamber and a reference object with a known area.

| **Image of Goryaev chamber** | | |
|---|---|---|
| **x112** | **x350** | **x700** |
| 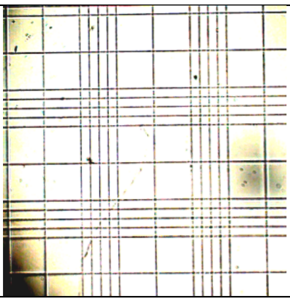 | 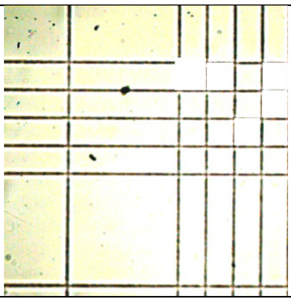 | 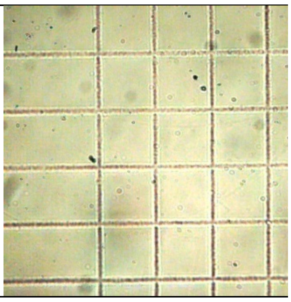 |
| The square of the Goryaev chamber with a known area of 0.0025 mm$^2$ | | |
| 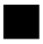 | 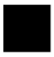 | 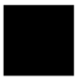 |

The images of the reference objects were processed in the program "Spectrum of differential distribution". It is estimated that these objects at magnifications x110, x350, x700 occupy an area of 256, 1936, 8836 pixels, respectively.

Further calculations were reduced to finding the equivalent diameter of a circle that occupies the same area. For equal values of the area of a circle and a square, the ratio of the side of the square to the diameter of the circle is a constant value - 0.886. Therefore, the equivalent diameter of a particle with a known area expressed in pixels and a constant multiplicity of magnification can be described by a dependence of the form:

$$d = \frac{\sqrt{\dfrac{0.0025 \cdot Sp}{P}}}{0.886} \qquad \text{(S1)}$$

where, $S_P$ is the area of the particle, expressed in pixels;
0.0025 – the area of the large square of the Goryaev chamber, $mm^2$;
P – the area of the small square of the Goryaev chamber, pixel;
0.886 –constant

The expression (S1), taking into account mathematical transformations, can be represented as:
at magnification x110

$$d = 3.527 \cdot \sqrt{Sp}, \qquad (S2)$$

at magnification x350

$$d = 1.283 \cdot \sqrt{Sp}, \qquad (S3)$$

at magnification x700

$$d = 0.6 \cdot \sqrt{Sp}, \qquad (S4)$$

where, 3.527; 1.283; 0.6 are constants for corresponding constant magnification conditions, having the dimension of microns/pixel$^{1/2}$.

In general terms

$$d = K \cdot \sqrt{S_p} \qquad (S5)$$

After approximating the graphical dependence of the constant K on the magnification of the computer-microscopic setup, a one-parameter equation ($R > 0.99$) is obtained, which has the form:

$$K = 331.996 \cdot X^{-0.9585}, \qquad (S6)$$

where, X is the magnification of the computer-micro-optical installation

Combining equations (S5) and (S6), we obtained a dependence represented by equation (S7), which allows us to transform the linear particle sizes expressed in pixels into metric ones, which will take the form:

$$d = 331.996 \cdot X^{-0.9585} \cdot \sqrt{S_P}, \qquad (S7)$$

where, X is the magnification of the computer-micro-optical installation
$S_P$ – the area of the particle, expressed in pixels.
